# Morphometric study of third-instar larvae from five morphotypes of the *Anastrepha fraterculus* cryptic species complex (Diptera, Tephritidae)

**DOI:** 10.3897/zookeys.540.6012

**Published:** 2015-11-26

**Authors:** Nelson A. Canal, Vicente Hernández-Ortiz, Juan O. Tigrero Salas, Denise Selivon

**Affiliations:** 1 Universidad del Tolima, Barrio Altos de Santa Helena, Ibagué, Tolima, Colombia, CP 73000629; 2 Instituto de Ecología A.C., Red de Interacciones Multitróficas. Carretera antigua a Coatepec # 351, El Haya. Xalapa, Veracruz 91070, México; 3 Universidad de las Fuerzas Armadas, Departamento Ciencias de la Vida, Carrera de Ciencias Agropecuarias (IASA I), Laboratorio de Entomología, PO Box 171-5-231-B, Sangolquí, Ecuador; 4 Departamento de Biologia, Instituto de Biociências, Universidade de São Paulo, 05508-900 São Paulo, São Paulo, Brazil

**Keywords:** South American fruit fly, immature, taxonomy, geometric morphometry, linear morphometry, morphotypes

## Abstract

The occurrence of cryptic species among economically important fruit flies strongly affects the development of management tactics for these pests. Tools for studying cryptic species not only facilitate evolutionary and systematic studies, but they also provide support for fruit fly management and quarantine activities. Previous studies have shown that the South American fruit fly, *Anastrepha fraterculus*, is a complex of cryptic species, but few studies have been performed on the morphology of its immature stages. An analysis of mandible shape and linear morphometric variability was applied to third-instar larvae of five morphotypes of the *Anastrepha fraterculus* complex: Mexican, Andean, Ecuadorian, Peruvian and Brazilian-1. Outline geometric morphometry was used to study the mouth hook shape and linear morphometry analysis was performed using 24 linear measurements of the body, cephalopharyngeal skeleton, mouth hook and hypopharyngeal sclerite. Different morphotypes were grouped accurately using canonical discriminant analyses of both the geometric and linear morphometry. The shape of the mandible differed among the morphotypes, and the anterior spiracle length, number of tubules of the anterior spiracle, length and height of the mouth hook and length of the cephalopharyngeal skeleton were the most significant variables in the linear morphometric analysis. Third-instar larvae provide useful characters for studies of cryptic species in the *Anastrepha fraterculus* complex.

## Introduction

Some species within the Tephritidae family are among the most important pests for agriculture because of their direct effects on fruit production and the quarantine restrictions imposed to prevent the transfer of foreign species from one region to another (Schutze et al. 2012, Norrbom et al. 2013). In this family, there are species of agricultural importance that are, in reality, complexes of cryptic species (Kitthawee and Dujardin 2010, Hernández-Ortiz et al. 2012, Ruiz-Arce et al. 2012, Schutze et al. 2012, Krosch et al. 2013, Vaníčková et al. 2014). The occurrence of cryptic species among economically important fruit flies strongly affects the development of management tactics for these pests. Their economic importance is variable from one region to another, which makes the establishment of management practices more difficult. Detailed knowledge of the biology and taxonomy of these species is essential for the application of methods such as the sterile insect technique (SIT), the use of pheromones, the determination of pest-free or low-prevalence areas and quarantine measures or risk analysis (Frías et al. 2006, Schutze et al. 2012, Krosh et al. 2013, Norrbom et al. 2013, Perre et al. 2014).

The definition and determination of species is one of the most important topics in modern systematics. Traditionally, the description of species has been based on the study of morphological characteristics. In recent decades, other biological, ecological, genetic and evolutionary tools have been integrated with morphology to find new species, particularly within cryptic species complexes (Baylac et al. 2003, Bickford et al. 2007, Wiens 2007, de Queiroz 2007, Yeates et al. 2011, Krosh et al. 2013). Tools for studying cryptic species not only facilitate evolutionary and systematic studies, but they also provide support for fruit fly management and quarantine activities.

The South American fruit fly, *Anastrepha fraterculus* (Wiedemann), is a species of great economic importance within the genus and is subject to quarantine restrictions. It is widely distributed in America and is associated with a large number of host fruits (Hernández-Ortiz et al. 2012, Norrbom et al. 2013). In fact, this nominal species comprises a cryptic species complex, as has been demonstrated by genetic (Steck 1991, Steck and Sheppard 1993, Smith-Caldas et al. 2001) and cytogenetic (Selivon et al. 2004, 2005, Goday et al. 2006) studies, reproductive isolation tests (Selivon et al. 1999, Vera et al. 2006, Cáceres et al. 2009, Devescovi et al. 2014), chemo-taxonomy (Cáceres et al. 2009, Břízová et al. 2013, Vaníčková et al. 2015) and morphological (Selivon and Perondini 1998, Selivon et al. 2005, Hernández-Ortiz et al. 2004, 2012) analysis. Based on adult morphology, Hernández-Ortiz et al. (2012) identified seven morphotypes within this complex: Mexican, Andean, Venezuelan, Peruvian, and three morphotypes from the Brazilian territory, one of which extends to Argentina. In addition to these, Hernández-Ortiz et al. (2015) recently identified the Ecuadorian morphotype.

Studies of the immature stages may be informative for the definition of species limits as well as for studies of phylogeny and evolution (Norrbom et al. 1999, Dujardin et al. 2014). In addition, in the case of fruit flies these studies could be important for quarantine actions because this is the stage that damages fruits (Steck et al. 1990, Frías et al. 2008, Dutra et al. 2012) and the one that is mostly intercepted during trade. According to Frías et al. (2008), larvae of only 7% of Tephritidae species have been described in 17% of the genera. Studies on the larval morphology of *Anastrepha* have been performed by Steck and Malavasi (1988), Steck and Wharton (1988), Carroll and Wharton (1989), Steck et al. (1990), Frías et al. (2006, 2008, 2009) and Dutra et al. (2012). However, previous studies have barely covered the morphological descriptions of the studied species, except that of Steck et al. (1990), who used multivariate analysis to find traits which could separate 13 species. Frías et al. (2006, 2008) also studied larval differentiation among the genera *Anastrepha*, *Ceratitis*, *Bactrocera*, *Rhagoletis* and *Toxotrypana*; further, they differentiated the larvae of some species of *Rhagoletis* that occur in Chile. In larvae of fruit flies, only allometric studies have been performed. These studies have shown that several structures, such as the cephalopharyngeal skeleton and the mouth hook may have taxonomical importance for the group. However, the results have not been completely satisfactory.

The study of larvae would benefit from more sophisticated tools for measuring the extant morphologic variability, as could be the case of shape analysis of certain structures, since forms are among the features that show differences in the speciation processes (Jirakanjanakit et al. 2008, Schutze et al. 2012, Dujardin et al. 2014). Shape analysis through the study of outlines has been successfully applied to delimit cryptic species of mosquitoes and ticks (Dujardin et al. 2014) and to study the effect of hybridization in mandibles of stag beetles (Tatsuta et al. 2011). However, in spite of its capacity to detect minimal morphological variation, measurement errors can be introduced in geometric morphometric studies due to observer error, common in many works, photographing and collecting landmarks (Dujardin et al. 2010, Toro et al. 2010). Several solutions to this have been proposed (Arnqvist and Martensson 1998) with modern techniques of digital photography providing an adequate resolution for these liabilities (Dujardin et al. 2010).

The aim of this study was to perform a comparative analysis of third instar larvae of representatives of five morphotypes of the *Anastrepha fraterculus* complex (Mexican, Andean, Peruvian, Brazilian-1 and Ecuadorian). Through the use of geometric morphometry of the shape of the mouth hook and linear morphometry of larvae, we tested several variables and determined their usefulness in the differentiation of these morphotypes.

## Methods

*Biological material*. The taxonomic identity of all larvae used in this study was fully known from associated reared adults and the diagnoses developed by Hernández-Ortiz et al. (2004, 2012, 2015) (Table 1). The samples from Mexico and Ecuador derived from natural populations. The samples from Colombia and Brazil came from colonies reared for a few generations on host fruit in laboratory conditions. The sample from Peru came from a laboratory colony maintained on an artificial diet since 2002 at the laboratories of the International Atomic Energy Agency, in Seibersdorf, Austria. The sample of the Brazilian-1 morphotype was collected in the field and identified by one of the co-authors (DS) in the same location at which previous genetic, cytogenetic and morphometric studies were conducted with adults of this morphotype (Yamada and Selivon 2001). For each population we studied a total of 20 individuals.

**Table 1.** Data on collection of third-instar larvae of five morphotypes of the *Anastrepha fraterculus* complex.

Morphhotype	Country	State	Municipality	Host	Latitude	Longitude	Altitude
Andean	Colombia	Boyaca	Duitama	Guava feijoa (*Acca sellowiana*)	5°49'29,9"N	73°04'29,7"W	2569
Brazil sp1	Brazil	São Paulo	Itaquera	Guava (*Psidium guajaba*)	23°30'S	46°40'W	700
Ecuadorian	Ecuador	Pichincha	Quito	Custard apple (*Annona cherimola*)	00°06'47"S	78°25'33"W	1861
Mexican	Mexico	Veracruz	Teocelo	Guava	19°23'8"N	96°58'20"W	1190
Peruvian	Peru	Lima	La Molina	Custard apple	12°00'03"S	76°57´00"W	255

*Preparation of larvae*. Larvae were prepared following methods described by Frías et al. (2006) as follows: third-instar larvae were killed in boiling water for one minute in groups of up to 20 individuals and then put in a 75% alcohol solution for storage. Larvae specimens were photographed in dorsal view before proceeding with their preparation. Next, the larvae were left for one night in a 10% KOH solution, and the internal body content was withdrawn. Later, the cephalopharyngeal skeleton was carefully separated, removing the adhering tissue as much as possible. This structure was positioned in lateral view on a concave glass slide, slightly immersed in glycerin, covered and photographed. Digital images were also taken of the anterior spiracles by placing the cuticle on a glass slide with glycerin. The larval cuticle and the cephalopharyngeal skeleton were stored in Eppendorf tubes with glycerin and deposited in the Museum of the Laboratory of Entomology at the University of Tolima.

The left mouth hook was carefully separated, and the remaining tissue was removed as much as possible. Permanent slides were made with Canada balsam, putting the mandible in lateral view, and were deposited in the Museum of the Laboratory of Entomology at the University of Tolima. The mounting were done placing small amounts of Canada balsam each time to keep the mouth hooks in the best position to minimize the error.

*Image capture*. All pictures were taken with a Moticam10 digital camera, coupled to an Advance Optical stereoscopic microscope for digital images of the body, and a Carl Zeiss Primo Star Trinocular microscope was used for pictures of the mouthparts. In both cases, the camera had a 10X lens. The cephalopharyngeal skeleton and the anterior spiracle were photographed with a 10X objective, and the hypopharyngeal sclerite and mouth hook were photographed with a 40X objective. All digital images were taken at high resolution (3,664 × 2,748 pixels). The mouth hook at 400× magnification resulted in a 3D figure with blurred edges; therefore, multiple shots (between six and 10) were taken at different focal planes and later assembled with the software Helicon Focus 6.0.18 (2013). All the images were edited with Adobe Photoshop CS5 Extended 12.0 x64 (Adobe 2010). The third dimension can be ignored in geometric morphometry when it is not important compared to the other two, and if the imaged structure is in approximately the same position and of good quality (Zelditch et al. 2004, Dujardin et al. 2014). These methods minimized the variability of the data.

*Outline Geometric Morphometry*. The assessment of the shape variation of the mouth hook among the samples was performed using an elliptical Fourier analysis (EFA) (Tatsuta et al. 2011, Dujardin et al. 2014), for which points were marked on the image, making a complete outline description. Several modules of the CLIC software, version 84 (Dujardin 2013) were used in the analyses. The COO module was used for collecting the outlines, TET for concatenating the files, FOG for analysis and validation of classifications, and PAD to estimate the repeatability of the size and shape. Landmark captures were performed four times by a single observer (NA Canal) following Dujardin et al. (2010).

*Linear morphometry*. Samples were compared with a discriminant function analysis (DFA) applied over either linear measures between two points or the ratio between them. Measurements suggested by Steck and Wharton (1989), Steck et al. (1990) and Frías et al. (2006, 2008) were followed, and additional variables were included, which were deduced from the geometric morphometry study. We follow the terminology used by White et al. (1999) and Frías et al. (2008).

**Figure 1.**
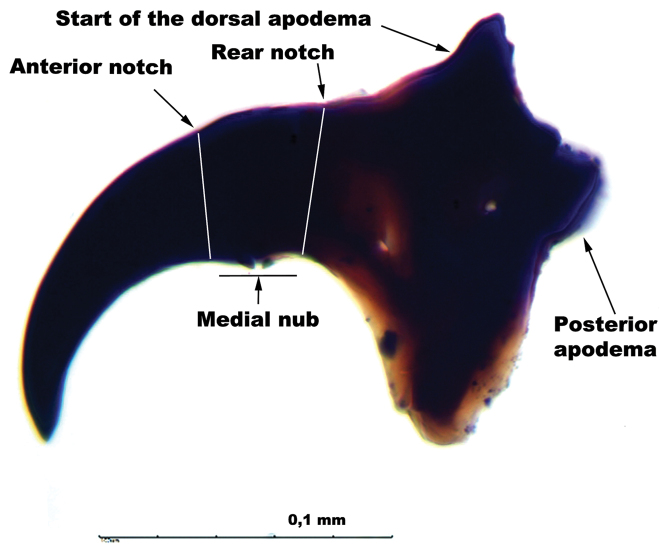
Lateral view of mouth hook of the third instar larvae of *Anastrepha fraterculus* complex.

The mouth hook morphology was observed carefully. Its shows a medial nub in the ventral curve, where the cuticle and muscles attaches, with a front and a rear notches next to it that extend to the top; a posterior apodema, like a neck, is also found. The anterior part of the dorsal apodema could be found where the slope turns greater (Figure 1).

**Figure 2.**
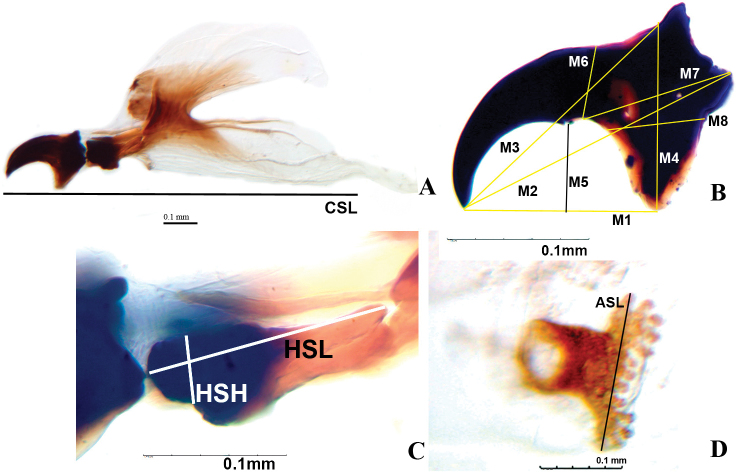
Linear variables measured in the third-instar larvae of the *Anastrepha fraterculus* complex. **A** cephalopharyngeal skeleton **B** mouth hook **C** hypopharyngeal sclerite **D** anterior spiracle. Variables are defined in the text.

All measurements were done on the digitized images of the structures. After variables were defined, measurements were performed three times by a single observer (NA Canal), but no differences in outcomes were found. Twenty-four variables were used, 15 of which corresponded to linear measurements, and nine to the ratios between various pairs (Figure 2).

### Abbreviations of the variables used are as follows

BL: body length; BW: body width at the sixth abdominal tergite; CSL: cephalopharyngeal skeleton length, from the anterior apex of the mandible to the end of the ventral cornua, at lower end of the dorsal cornua; HSL: hypopharyngeal sclerite length, from mouth hook joint to the rear distal point; and HSH: height of the hypopharyngeal sclerite at the anterior base of the hypopharyngeal bridge, perpendicular to the upper edge. The measurements of the mouth hook were M1: length from the apex to the ventral apodeme, M2: length from the apex to the dorsal most tip of neck, M3: length from the apex to the anterior base of the dorsal apodeme, M4: height from the apex of the ventral apodeme to the anterior base of the dorsal apodeme, M5: depth of ventral concavity from line M1 to tip of nub, M6: thickness of mouthhook at posterior base of nub by the posterior notch, M7: distance between the posterior base of nub and dorsal most tip of neck, and M8: width of the ventral apodeme at the base of the neck, in a line parallel to M1. ASL: width of the left anterior spiracle between the apices of the most extreme tubules, AST: number of tubules of the anterior spiracles, X1: BL/BW, X2: M1/M4, X3: M2/M4, X4: M1/M5, X5: M2/M5, X6: M3/M4, X7: CSL/HSL, X8: CSL/M3, and X9: CSL/M1.

*Data analysis*. The shape of the mouth hook was studied with an outline analysis in a two-dimensional plane, for which an EFA (Tatsuta et al. 2011, Dujardin et al. 2014) was used. Briefly, the outline curve was decomposed into a series of ellipses based on their sine and cosine; each one was referred to as a harmonic, and each harmonic was represented by four coefficients (Fourier coefficients). Based on the coefficients of the first harmonic, the rest of the coefficients were standardized to be used in later analyses. EFAs require doing principal components analysis (PCA) on the standardized coefficients. Based on the first principal components obtained, a DFA was performed, and afterwards, each individual was reclassified through a Jackknife procedure.

For the linear morphometry, a multivariate analysis was performed. The mean and standard deviations were calculated, and normality and homogeneity of variance tests were run for each of the variables. To assess the probability of individuals being classified into the predicted groups defined by the morphotypes and the contribution of each of the variables for group discrimination, a DFA was performed on the complete dataset, with the forward stepwise method. A canonical analysis was done to determine the canonical variables and their significance through a Chi-squared test. All analyses were performed using Statistica 12 (StatSoft 2014).

## Results

**Figure 3.**
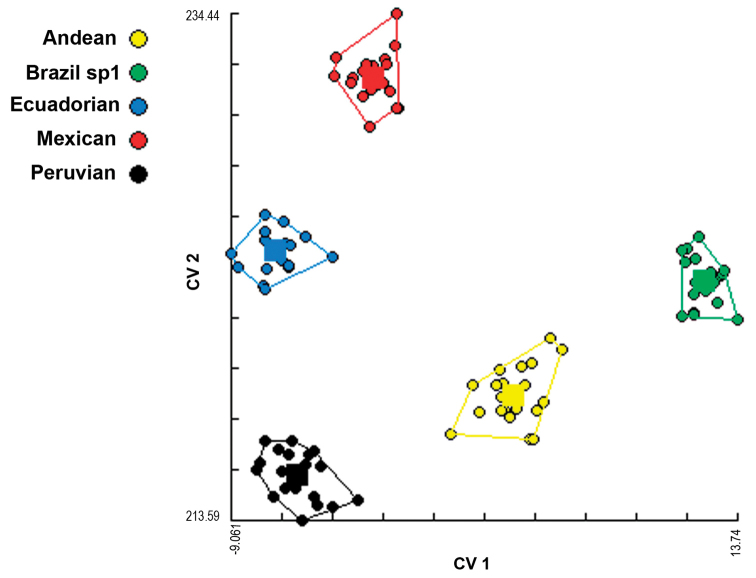
Grouping analysis of five morphotypes of the *Anastrepha fraterculus* complex, according to the shape of the mouth hook of third-instar larvae based on the values of the first two canonical factors in the discriminant analysis. The contribution of the first factor was 44%, and that of the second was 27%.

**Figure 4.**
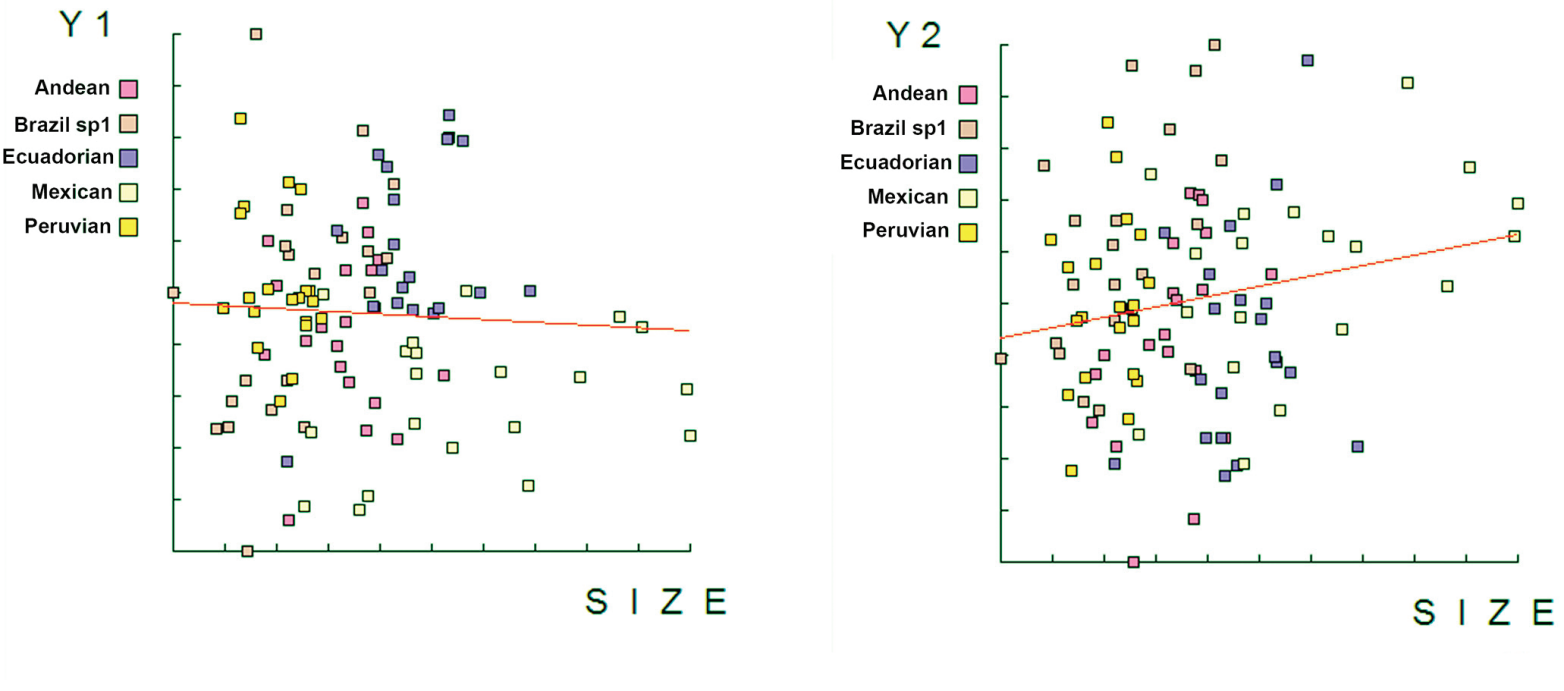
Allometric study indicating the influence of the mouth hook size in grouping five morphotypes of the *Anastrepha fraterculus* complex, studied with an elliptical Fourier analysis.

*Mouth hook shape*. The discriminant function analysis showed that all the samples studied differed in the shape of the mouth hook (Figure 3). The analysis of reclassification of the individuals correctly included 100% of the individuals into the expected morphotype. The allometric analysis showed a 0% influence of the size on Canonical Factor 1, and a 3% influence on Canonical Factor 2, indicating that the size of the individuals did not influence the results on the shape of the mouth hook (Figure 4).

**Figure 5.**
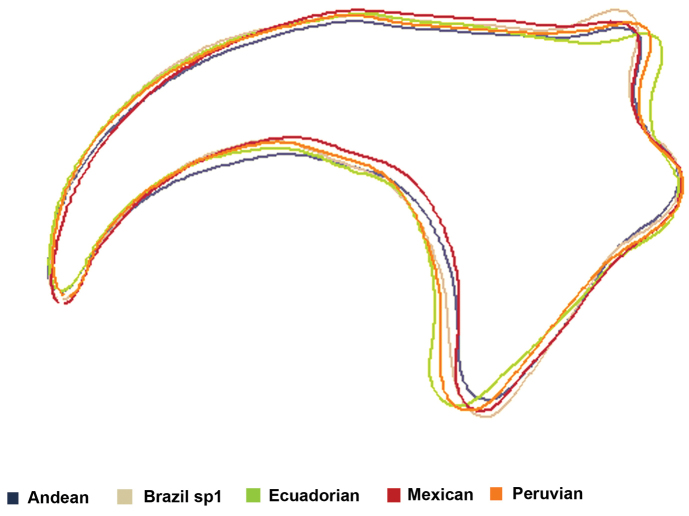
Representative outline of the mouth hook shape in third-instar larvae of five morphotypes of the *Anastrepha fraterculus* complex, obtained through an elliptical Fourier analysis.

The mouth shape outlines for each individual were aligned, rotated and grouped to build the representative shapes of the morphotypes (Figure 5). The morphotypes showed variability mainly over the dorsal and ventral apodemes and, less noticeably, over the width of the middle part.

*Size variability of the individuals*. The variability of the individual sizes was studied through the morphometry of the larvae. The DFA included all 18 variables of the model (excluding CSL, M2, X4, X6, X7, and X8); 10 of the variables resulted in statistically significant differences for the segregation of the morphotypes (Wilks’ Lambda: 0.005 approx. F(72,309)=12.224, p<0.0001) (Table 2). The statistically significant variables were the body length and width of the larvae, three measurements of the mouth hook, the hypopharyngeal sclerite, and the size and number of the anterior spiracle tubules. The average body length (BL) was greater in the Peruvian, Ecuadorian and Mexican morphotypes, and body width (BW) was greater in the Andean morphotype; the ratio between these two measurements (X1) was lower in the Andean population. The HSL was, on average, longer in the Andean and Ecuadorian morphotypes. The mouth hook length between the apex and the ventral apodeme (M1) was shorter for the Peruvian morphotype; the mean width in the middle part of the mouth hook (M6) was greater for individuals of the Ecuadorian morphotype and smallest for the Peruvian morphotypes. The average basal width of the ventral apodeme (M8) was narrower in specimens of the Brazilian-1 and Peruvian morphotypes. The anterior spiracle was longer in specimens of the Andean morphotype, and the number of spiracle tubules was smaller in the Brazilian-1 and Peruvian morphotypes. The X9 ratio between the CSL and the length of the mouth hook from the apex to the ventral apodeme (M1) was greater for Peruvian individuals and smaller for Mexican individuals (Table 3). The standard deviations of the measurements were low (Table 3), indicating that accuracy of measurements was high and repeatable.

**Table 2.** Independent contribution to the discriminant model of each of the variables measured from third-instar larvae of five morphotypes of the *Anastrepha fraterculus* complex. * < 0.05 = statistically significant.

Variables	Wilks’ Lammbda	F-remove (4.78)	p-value
ASD	0.006756	6.85482	<0.0001*
M5	0.005493	1.92970	0.113783
ASL	0.008777	14.74068	<0.0001*
X9	0.006039	4.05858	0.004*
M6	0.005942	3.67924	0.008*
X1	0.006071	4.18342	0.004*
M3	0.005450	1.76149	0.145094
X3	0.005190	0.74581	0.563734
M1	0.005842	3.29114	0.015*
HSL	0.005989	3.86235	0.006*
HSH	0.005509	1.99152	0.103994
X2	0.005472	1.84521	0.128604
M8	0.006221	4.76893	0.001*
M4	0.005597	2.33372	0.062912
M7	0.005471	1.84152	0.129291
BL	0.006709	6.67357	0.0001*
BW	0.006513	5.90854	0.0003*
X5	0.005293	1.14799	0.340365

**Table 3.** Means and standard deviations of 24 morphometric variables of third-instar larvae of five morphotypes of the *Anastrepha fraterculus* complex. * Statistical difference.

Population	BL	BW	HSL	HSH	CSL	M1	M2	M3	M4	M5	M6	M7	M8	X1	X2	X3	X4	X5	X6	ASL	ASD	X7	X8	X9	Valid N
Andean	9.22±0.97	2.09±0.19	0.2±0.015	0.07±0.006	1.14±0.07	0.17±0.01	0.25±0.01	0.23±0.01	0.15±0.016	0.08±0.007	0.06±0.008	0.1±0.007	0.06±0.008	4.41±0.31	1.14±0.10	1.69±0.15	2.21±0.24	3.26±0.32	1.55±0.10	0.34±0.07	14.45±0.89	5.79±0.32	4.96±0.39	6.72±0.49	20
BrasilSp1	8.77±0.74	1.71±0.29	0.17±0.012	0.07±0.007	1.05±0.04	0.15±0.02	0.23±0.02	0.21±0.02	0.14±0.016	0.08±0.006	0.05±0.007	0.09±0.006	0.05±0.005	5.23±0.72	1.07±0.08	1.59±0.11	2.03±0.26	3±0.31	1.44±0.09	0.19±0.02	10.55±0.83	6.09±0.45	5.14±0.36	6.91±0.70	20
Peruvian	9.48±0.38	1.87±0.14	0.18±0.007	0.07±0.005	1.09±0.04	0.14±0.01	0.22±0.01	0.21±0.01	0.14±0.008	0.08±0.005	0.05±0.004	0.09±0.007	0.06±0.006	5.1±0.34	1±0.07	1.56±0.09	1.9±0.17	2.94±0.21	1.46±0.07	0.2±0.02	11.55±1.0	6.09±0.25	5.19±0.28	7.56±0.45	20
Ecuador	10.02±0.37	1.89±0.13	0.21±0.013	0.07±0.008	1.15±0.04	0.16±0.01	0.26±0.01	0.25±0.01	0.16±0.012	0.08±0.006	0.06±0.006	0.1±0.006	0.07±0.007	5.32±0.31	1.01±0.09	1.59±0.11	1.98±0.16	3.12±0.23	1.51±0.09	0.24±0.02	14.1±1.12	5.47±0.28	4.68±0.24	7.02±0.48	20
Mexico	9.7±0.73	2±0.17	0.19±0.026	0.07±0.009	1.15±0.11	0.18±0.03	0.27±0.03	0.25±0.03	0.17±0.023	0.09±0.008	0.06±0.011	0.11±0.014	0.06±0.010	4.86±0.32	1.07±0.05	1.6±0.07	1.93±0.22	2.89±0.23	1.49±0.09	0.23±0.03	12.75±1.02	5.98±0.31	4.55±0.24	6.34±0.51	20
All Grps	9.44±0.79	1.91±0.23	0.19±0.02	0.07±0.007	1.12±0.08	0.16±0.02	0.25±0.03	0.23±0.02	0.15±0.019	0.08±0.009	0.06±0.009	0.1±0.011	0.06±0.009	4.98±0.54	1.06±0.09	1.61±0.12	2.01±0.24	3.04±0.29	1.49±0.10	0.24±0.06	12.68±1.77	5.88±0.40	4.9±0.39	6.91±0.66	100

**Figure 6.**
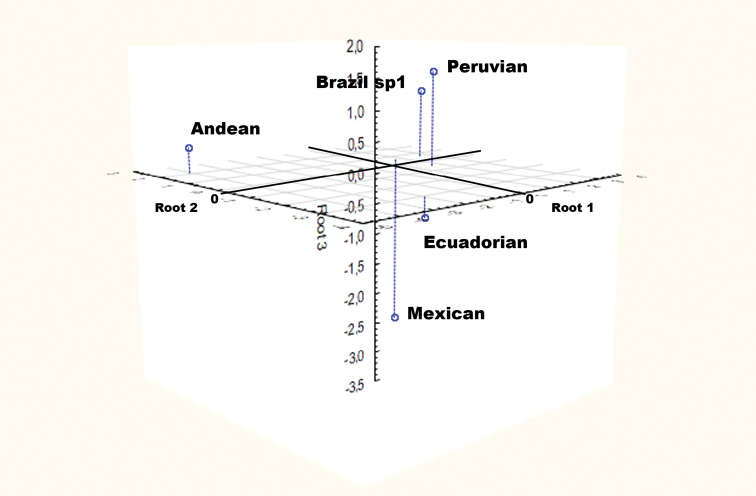
3D scatterplot of discriminant function analysis applied to the centroid values of 24 measurements in third-instar larvae of five morphotypes of the *Anastrepha fraterculus* complex.

The canonical analysis resulted in four canonical roots, and the Chi-squared test showed statistical significance for all the roots. CV-1 had 51.6% of the discrimination power, CV-2 had 24.2%, CV-3 had 19.3% and CV-4 had 4.9%. In the first root, variables with major contribution to the separation of the groups were the anterior spiracle length (ASL) and the number of tubules of the anterior spiracle (AST), followed in importance by the hypopharyngeal sclerite length (HSL), body width (BW), and dimensions of the mouth hook M3 (length from the apex to the most distal and dorsal point) and M8 (width of the ventral apodeme). The most important variables for CV-2 were the ASL, BL/BW (X1) and mouth hook length/width (X2), followed by the BL and HSL (Table 4). The 3D graph of the morphotype centroids, including the first three canonical roots, shows the separation of the different populations (Figure 6).

**Table 4.** Correlation between the variables and canonical roots from the discriminant analysis for 24 measurements of third-instar larvae of five morphotypes of the *Anastrepha fraterculus* complex.

Variable	Root 1	Root 2	Root 3	Root 4
ASD	-0.545921	-0.100158	-0.344710	0.172453
M5	0.041755	-0.071879	-0.653270	0.146808
ASL	-0.513976	0.354166	-0.104138	-0.004608
X9	0.036323	-0.175930	0.385942	0.368080
M6	-0.119046	-0.177843	-0.293162	-0.329549
X1	0.159367	-0.335778	0.090498	-0.231802
M3	-0.183020	-0.159496	-0.634346	0.078811
X3	-0.118893	0.134014	-0.037045	-0.103410
M1	-0.106642	0.111731	-0.475412	-0.141087
HSL	-0.254104	-0.211098	-0.239891	-0.017785
HSH	-0.071814	0.014497	0.099759	0.055553
X2	-0.103959	0.305811	-0.053167	-0.234816
M8	-0.189002	-0.150179	-0.247975	0.111262
M4	-0.038899	-0.119367	-0.407167	0.016389
M7	-0.077578	-0.007283	-0.429200	0.136471
BL	-0.075040	-0.250882	-0.204123	0.306914
BW	-0.182774	0.132194	-0.198878	0.344580
X5	-0.177914	0.039586	0.090199	-0.187916
Eigenvalue	6.9315	3.239	2.599	0.653
Cumulative proportion	0.5164	0.758	0.951	1.000

The prediction model indicated that 96% of the individuals were correctly placed in their respective morphotypes; all of the Andean and Ecuadorian specimens were properly classified, and only two individuals from Brazilian-1, one from Mexico and one from Peru were incorrectly classified (Table 5).

**Table 5.** Classification matrix of individuals according to a predictive model of third-instar larvae of five morphotypes of the *Anastrepha fraterculus* complex. Rows: Observed classifications; Columns: Predicted classifications. Same probabilities for all the groups.

Group	Percent	Andean	Brasil sp1	Peruvian	Ecuador	Mexico
Andean	100.0000	20	0	0	0	0
BrasilSp1	90.0000	0	18	2	0	0
Peruvian	95.0000	0	1	19	0	0
Ecuadorian	100.0000	0	0	0	20	0
Mexican	95.0000	0	1	0	0	19
Total	96.0000	20	20	21	20	19

## Discussion

The results obtained from our study of the mouth hook shape of the third-instar larvae established that variation exists in the shape of this structure that usefully separates the five morphotypes. Moreover, it was possible to confirm the presence of variability in the dorsal and ventral apodeme areas. Geometric morphometry is a sensitive tool to study the presence of cryptic species (Adams et al. 2004, Dujardin 2008, Dujardin et al. 2010, 2014), and recently, outline geometric morphometry has facilitated the study of complex structures, for example, in immature stages of insects (Dujardin et al. 2014). To the extent of our knowledge, this is the first study to use outline-based morphometrics for immature stages of tephritid fruit flies.

Geometric morphometry has been used for differentiation of fruit flies (Kitthawee and Dujardin 2010, Yee et al. 2011, Schutze et al. 2012, Perre et al. 2014, Hernández-Ortiz et al. 2015), in all cases on adult structures, leading to the possibility of differentiating formal species or cryptic species complexes. Unfortunately, geometric morphometry does not generate characters that can be used in traditional taxonomic keys, and further studies are needed to determine how to use this information (Dujardin et al. 2010). An alternative is, for example, the development of automatic systems for identification, as suggested by Faria et al. (2014), based on the geometric morphology of adults from several species of *Anastrepha*. The results obtained from our study suggest that the methods proposed here could be used for developing an identification system of this type that extends to larvae.

The results of the linear morphometry were also highly satisfactory, reaching a 96% accuracy of the predicted classification for the studied individuals. According to what was previously reported regarding size variation of insects through generations of laboratory rearing (Jaramillo et al. 2002, Jirakanjanakit et al. 2008), it is possible that these results were especially influenced by the long breeding of the Peruvian morphotype. Repeatability of results could be supposed based on the low variance of the variables.

Steck et al. (1990) used morphometric and morphological variables to differentiate 13 species of *Anastrepha* based on the study of third-instar larvae. The accuracy of the key produced was high for species differentiation, despite the high variability in some of the species. Samples of the nominal species *Anastrepha fraterculus* from various parts of Latin America were included in that study, and the authors found that it was one of the species with highest variability in the characters analyzed. Currently it is well known that, in reality, the authors studied different cryptic species, hence the difficulty in identifying *Anastrepha fraterculus* species. However, these authors could establish differences between species through normalization of variables by transformation and construction of linear discriminant functions. Our study included new variables to differentiate the cryptic species of *Anastrepha fraterculus*, which could complement the study of Steck et al. (1990) for recognizing species, by including additional morphological characters.

Still these techniques have some difficulties. The most common errors made in this analysis result from poor mounting of structures, digital imaging and determination of landmarks (Dujardin et al. 2010). The principal error in our study could be in the imprecision of the landmarks; however, after some testing, our approach resulted in a minimized error and in a consistent and reliable data for analysis. Outcomes of the classification of individuals and the low variance on linear variables of the model supported this conclusion.

Specimens studied here derive from different sources, either from wild samples reared on natural hosts or from lab strains reared on artificial diets, however we do not know the effects of this on the measured structures, and further studies are needed. Some authors have suggested that developmental conditions affect the size of insects (Jaramillo et al. 2002, Schutze et al. 2012). Jaramillo et al. (2002) and Jirakanjanakit et al. (2008) found variability in the head and wing size of insects reared in the laboratory for several generations, but not in the shape until after at least 10 generations. Our sample of the Peruvian morphotype came from a colony artificially reared since 2002, and may deviate from wild specimens. However the low values resulting from the allometric studies (CV-1=0%, CV-2=3%, Figure 4) confirm that our results are due to the shape but not the size of the individuals.

In many cases, the use of morphological characters of immature stages of insects for phylogenetic studies has helped to improve the understanding of relationships among groups (see revision in Meier and Lim 2009). Even though, immature stages have been widely ignored in studies of taxonomy, systematics and phylogeny, perhaps due to the difficulty of associating them with the adults and of determining stable morphological characters for them (Meier and Lim 2009). We suggest that further effort should be made in rearing specimens and revising methods and characters.

## Conclusions

Outline geometric morphometry and linear morphometry proved to be useful tools for the study of cryptic species of the *Anastrepha fraterculus* complex. The results obtained from this work with third-instar larvae should be expanded to include additional populations to strengthen the dataset and advance our tools to study cryptic species of economically important fruit flies.

## References

[B1] Adams DC Rohlf FJ Slice DE (2004) Geometric morphometrics: Ten years of progress following the “revolution”. Italian Journal of Zoology 71: 5–16. doi: 10.1080/11250000409356545

[B2] Adobe System Incorporated (2010) Adobe PhotoShop CS5 Extended version 12.0x64, 1990–2010.

[B3] Arnqvist G Mårtensson T (1998) Measurment error in geometrics morphometrics: empirical strategies to assess and reduce its impact on measures of shape. Acta Zoologica Academiae Scientiarum Hungaricae 44: 73–96.

[B4] Baylac M Villemant C Simbolotti G (2003) Combining geometric morphometrics with pattern recognition for the investigation of species complexes. Biological Journal of the Linnean Society 80: 89–98. doi: 10.1046/j.1095-8312.2003.00221.x

[B5] Bickford D Lohman DL Sodhi NS Ng PKL Meier R Winker K Ingram KK Das I (2007) Cryptic species as a window on diversity and conservation. Trends in Ecology and Evolution 22: 148–155. doi: 10.1016/j.tree.2006.11.004 17129636 10.1016/j.tree.2006.11.004

[B6] Břízová R Mendonça AL Vanícková L Mendonça AL Da Silva CE Tomčala A Paranhos BAJ Dias VS Joachim-Bravo IS Hoskovec M Kalinová B Nascimento RR (2013) Pheromone analyses of the *Anastrepha fraterculus* (Diptera: Tephritidae) cryptic species complex. Florida Entomologist 96: 1107–1115. doi: 10.1653/024.096.0351

[B7] Cáceres C Segura DF Vera MT Wornoayporn V Cladera JL Teal P Sapountzis P Bourtzis K Zacharopoulou A Robinson AS (2009) Incipient speciation revealed in *Anastrepha fraterculus* (Diptera; Tephritidae) by studies on mating compatibility, sex pheromones, hybridization and cytology. Biological Journal of the Linnean Society 97: 152–165. doi: 10.1111/j.1095-8312.2008.01193.x

[B8] Carroll L Wharton R (1989) Morphology of the immature stages of *Anastrepha ludens* (Diptera: Tephritidae). Annals of the Entomological Society of America 82: 201–214. doi: 10.1093/aesa/82.2.201

[B9] De Queiroz K (2007) Species concepts and species delimitation. Systematic Biology 56: 879–886. doi: 10.1080/10635150701701083 18027281 10.1080/10635150701701083

[B10] Devescovi F Abraham S Kelly A Nolazco N Castañeda MR Tadeo E Caceres C Segura D Vera T Joachim-Bravo I Canal NA Rull J (2014) Ongoing speciation within the *Anastrepha fraterculus* (Diptera: Tephritidae) cryptic species complex: the case of the Andean morphotype. Entomologia Experimentalis et Applicata 152: 238–247. doi: 10.1111/eea.12219

[B11] Dujardin JP (2008) Morphometrics applied to medical entomology. Infection, Genetics and Evolution 8: 875–890. doi: 10.1016/j.meegid.2008.07.011 10.1016/j.meegid.2008.07.011 18832048

[B12] Dujardin JP (2013) MoMe-CLIC: Morphometrics in Medical Entomology, Collection of Landmarks for Identification and Characterization. http://mome-clic.com

[B13] Dujardin JP Kaba D Henry AB (2010) The exchangeability of shape. BMC Research Notes 3: . doi: 10.1186/1756-0500-3-266 10.1186/1756-0500-3-266 PMC2987866 20964872

[B14] Dujardin JP Kaba D Solano P Dupraz M McCoy KD Jaramillo-O N (2014) Outline-based morphometrics, an overlooked method in arthropod studies? Infections, Genetics and Evolution 28: 704–714. doi: 10.1016/j.meegid.2014.07.035 10.1016/j.meegid.2014.07.035 25111609

[B15] Dutra VS Ronchi-Teles B Steck GJ Silva JG (2012) Description of larvae of *Anastrepha* spp. (Diptera: Tephritidae) in the *fraterculus* group. Annals of the Entomological Society of America 105: 529–538 doi: 10.1603/AN11180

[B16] Faria FA Perre P Zucchi RA Jorge LR Lewinsohn TM Rocha A Torres RS (2014) Automatic identification of fruit flies (Diptera: Tephritidae). Journal of Visual Communication and Image Representation 25: 1516–1527. doi: 10.1016/j.jvcir.2014.06.014

[B17] Frías D Hernández-Ortiz V Vaccaro NC Bartolucci AF Salles LA (2006) Comparative morphology of immature stages of some frugivorous species of fruit flies (Diptera: Tephritidae). Israel Journal of Entomology 35/36: 423–457.

[B18] Frías D Selivon D Hernández V (2008) Taxonomy of immature stages: new morphological characters for Tephritidae larvae identification. In: Sugayana R Zucchi RA Ovruski S Sivinski J (Eds) Fruit flies of economic importance: from basic to applied knowledge. 7th International Symposium on Fruit Flies of Economic Importance, Salvador, Brazil, September 2006 Press Color Gráficos Especializados Ltda, 29–44.

[B19] Frías D Hernández-Ortiz V López L (2009) Description of the third-instar of *Anastrepha leptozona* Hendel (Diptera: Tephritidae). Neotropical Entomology 38: 491–496. doi: 10.1590/S1519-566X2009000400008 19768267 10.1590/s1519-566x2009000400008

[B20] Goday C Selivon D Perondini ALP Greciano PG Ruiz MF (2006) Cytological characterization of sex chromosomes and ribosomal DNA location in *Anastrepha* species (Diptera, Tephritidae). Cytogenetic and Genome Research 114: 70–76. doi: 10.1159/000091931 16717453 10.1159/000091931

[B21] Helicon Focus (2013) Helicon Focus, version 6.0.18, 2000–2013. Helicon Soft Ltd. http://www.heliconsoft.com

[B22] Hernández-Ortiz V Gómez-Amaya JA Sánchez BA McPheron BA Aluja M (2004) Morphometric analysis of Mexican and South American populations of the *Anastrepha fraterculus* complex (Diptera: Tephritidae) and recognition of a distinct Mexican morphotype. Bulletin of Entomological Research 94: 487–499. doi: 10.1079/BER2004325 15541188 10.1079/ber2004325

[B23] Hernández-Ortiz V Bartolucci A Morales-Valles P Frias D Selivon D (2012) Cryptic species of the *Anastrepha fraterculus* complex (Diptera:Tephritidae): A multivariate approach for the recognition of South American morphotypes. Annals of the Entomological Society of America 105: 305–318. doi: 10.1603/AN11123

[B24] Hernández-Ortiz V Canal NA Tigrero Salas JO Ruíz-Hurtado FM Dzul-Cauich JF (2015) Taxonomy and phenotypic relationships of the *Anastrepha fraterculus* complex in the Mesoamerican and Pacific Neotropical dominions (Diptera, Tephritidae). In: De Meyer M Clarke AR Vera MT Hendrichs J (Eds) Resolution of Cryptic Species Complexes of Tephritid Pests to Enhance SIT Application and Facilitate International Trade. ZooKeys 540: 95–124. doi: 10.3897/zookeys.540.6027 10.3897/zookeys.540.6027 PMC4714066 26798256

[B25] Jaramillo N Castillo D Wolff M (2002) Geometric morphometric differences between *Panstrongylus geniculatus* from field and laboratory. Memorias do Instituto Oswaldo Cruz 97: 667–673. doi: 10.1590/S0074-02762002000500015 12219133 10.1590/s0074-02762002000500015

[B26] Jirakanjanakit N Leemingsawat S Dujardin JP (2008) The geometry of the wing of Aedes (Stegomyia) aegypti in isofemale lines through successive generations. Infection, Genetics and Evolution 8: 414–421. doi: 10.1016/j.meegid.2007.05.004 10.1016/j.meegid.2007.05.004 17600773

[B27] Kitthawee S Dujardin JP (2010) The geometric approach to explore the *Bactrocera tau* complex (Diptera: Tephritidae) in Thailand. Zoology 113: 243–249. doi: 10.1016/j.zool.2009.12.002 20817492 10.1016/j.zool.2009.12.002

[B28] Krosch NM Schutze MK Armstrong KF Boontop Y Boykin LM Chapman TA Englezou A Cameron SL Clarke AR (2013) Piecing together an integrative taxonomic puzzle: microsatellite, wing shape and aedeagus length analyses of *Bactrocera dorsalis* *s.l.* (Diptera: Tephritidae) find no evidence of multiple lineages in a proposed contact zone along the Thai/Malay Peninsula. Systematic Entomology 38: 2–13. doi: 10.1111/j.1365-3113.2012.00643.x

[B29] Meier R Lim GS (2009) Conflict, convergent evolution, and the relative importance of immature and adult characters in endopterygote phylogenetics. Annual Review of Entomology 54: 85–104. doi: 10.1146/annurev.ento.54.110807.090459 10.1146/annurev.ento.54.110807.090459 18817507

[B30] Norrbom AL Zucchi RA Hernández-Ortiz V (1999) Phylogeny of the genera *Anastrepha* and *Toxotrypana* (Trypetinae: Toxotrypanini) based on morphology. In: Aluja M Norrbom AL (Eds) Fruit Flies (Tephritidae): Phylogeny and Evolution of Behavior. CRC Press, Boca Raton, 299–341.

[B31] Norrbom AL Korytkowski CA Zucchi RA Uramoto K Venable GL McCormick J Dallwitz MJ (2013) *Anastrepha* and *Toxotrypana*: descriptions, illustrations, and interactive keys. Version: 28^th^ September 2013. http://delta-intkey.com/anatox/citation.htm

[B32] Perre P Jorge LR Lewinsohn TM Zucchi RA (2014) Morphometric differentiation of fruit fly pest species of the *Anastrepha fraterculus* group (Diptera: Tephritidae). Annals of the Entomological Society of America 10: 490–495. doi: 10.1603/AN13122

[B33] Ruiz-Arce R Barr N Owen CL Thomas DB McPheron BA (2012) Phylogeography of *Anastrepha obliqua* inferred with mtDNA sequencing. Journal of Economic Entomology 105: 2147–2160. doi: 10.1603/EC1221 23356081 10.1603/ec12211

[B34] Schutze MK Jessup A Clarke AR (2012) Wing shape as a potential discriminator of morphologically similar pest taxa within the *Bactrocera dorsalis* species complex (Diptera: Tephritidae). Bulletin of Entomological Research 102: 103–111. doi: 10.1017/S0007485311000423 21867577 10.1017/S0007485311000423

[B35] Selivon D Perondini ALP (1998) Eggshell morphology in two cryptic species of *Anastrepha fraterculus* (Diptera: Tephritidae). Annals of the Entomological Society of America 91: 473–478. doi: 10.1093/aesa/91.4.473

[B36] Selivon D Perondini ALP Morgante JS (1999) Haldane’s rule and other aspects of reproductive isolation observed in the *Anastrepha fraterculus* complex (Diptera: Tephritidae). Genetics and Molecular Biology 22: 507–510. doi: 10.1590/S1415-47571999000400007

[B37] Selivon D Vretos C Fontes L Perondini ALP (2004) New variant forms in the *Anastrepha fraterculus* complex (Diptera: Tephritidae). In: Barnes BN (Ed.) Proceedings of the 6^th^ International Symposium of Fruit Flies of Economic Importance, Stellenbosch, South Africa, May 2002 Isteg Scientific Publications, Irene-South Africa, 253–258.

[B38] Selivon D Perondini ALP Morgante JS (2005) A genetic-morphological characterization of two cryptic species of *Anastrepha fraterculus* complex (Diptera, Tephritidae). Annals of the Entomological Society of America 98: 367–381. doi: 10.1603/0013-8746(2005)098[0367:AGCOTC]2.0.CO;2

[B39] Smith-Caldas MRB McPheron BA Silva JG Zucchi RA (2001) Phylogenetic relationships among species of the *fraterculus* group (*Anastrepha*: Diptera: Tephritidae) inferred from DNA sequences of mitochondrial Cytochrome Oxidase I. Neotropical Entomology 30: 565–573. doi: 10.1590/S1519-566X2001000400009

[B40] StatSoft Inc (2014) Statistica Software for statistical analysis, version 12, 1984–2014.

[B41] Steck GJ (1991) Biochemical systematics and population genetic structure of *Anastrepha fraterculus* and related species (Diptera: Tephritidae). Annals of the Entomological Society of America 84: 10–28. doi: 10.1093/aesa/84.1.10

[B42] Steck GJ Malavasi A (1988) Description of the immature stages of *Anastrepha bistrigata* (Diptera:Tephritidae). Annals of the Entomological Society of America 81: 1004–1009. doi: 10.1093/aesa/81.6.1004

[B43] Steck G Wharton A (1988) Description of immature stages of *Anastrepha interrupta*, *A. limae*, and *A. grandis* (Diptera: Tephritidae). Annals of the Entomological Society of America 81(6): 994–1003. doi: 10.1093/aesa/81.6.994

[B44] Steck GJ Sheppard WS (1993) Mitochondrial DNA variation in *Anastrepha fraterculus*. In: Aluja M Liedo P (Eds) Fruit Flies: Biology and Management. Springer Verlag, New York, 9–14. doi: 10.1007/978-1-4757-2278-9_2

[B45] Steck G Carroll L Celedonio H Guillen J (1990) Methods for identification of *Anastrepha* larvae (Diptera: Tephritidae), and key to 13 species. Proceedings of the Entomological Society of Washington 92: 333–346.

[B46] Tatsuta H Iwata H Goka K (2011) Morphometric studies of male Lucanid beetle mandibles: shape variation between hybrid subspecies. In: Lestrel PE (Ed.) Biological Shape Analysis. 1st International Symposium on Biological Shape Analysis, Okinawa, Japan, June 2009 World Scientific Publishing, Singapore, 87–103. doi: 10.1142/9789814355247_0007

[B47] Toro IMV Manríquez SG Suazo GI (2010) Morfometría geométrica y el estudio de las formas biológicas: de la morfología descriptiva a la morfología cuantitativa. International Journal of Morphology 28: 977–990. doi: 10.4067/S0717-95022010000400001

[B48] Vaníčková L Virgilio M Tomčala A Břízová R Ekesi S Hoskovec M Kalinová B Nascimento RR do De Meyer M (2014) Resolution of three cryptic agricultural pests (*Ceratitis fasciventris*, *C. anonae*, *C. rosa*, Diptera: Tephritidae) using cuticular hydrocarbon profiling. Bulletin of Entomological Research 104: 631–638. doi: 10.1017/S0007485314000406 24896539 10.1017/S0007485314000406

[B49] Vaníčková L Břízová R Mendonça AL Pompeiano A Nascimento RR do (2015) Intraspecific variation of cuticular hydrocarbon profiles in the *Anastrepha fraterculus* (Diptera: Tephritidae) species complex. Journal of Applied Entomology. doi: 10.1111/jen.12204

[B50] Vera MT Cáceres C Wornoayporn V Islam A Robinson AS De La Vega MH Hendrichs J Cayol JP (2006) Mating incompatibility among populations of the South American fruit fly *Anastrepha fraterculus* (Diptera: Tephritidae). Annals of the Entomological Society of America 99: 387–397.

[B51] White IM Headrick DH Norrbom AL Carroll LE (1999) Glossary. In: Aluja M Norrbom AL (Eds) Fruit Flies (Tephritidae): Phylogeny and evolution of behavior. CRC Press, Boca Raton, FL, 881–924.

[B52] Wiens JJ (2007) Species delimitation: New approaches for discovering diversity. Systematic Biology 56: 875–878. doi: 10.1080/10635150701748506 18027280 10.1080/10635150701748506

[B53] Yamada MS Selivon D (2001) Rose, an eye color mutation in a species of the *Anastrepha fraterculus* complex (Diptera: Tephritidae). Annals of the Entomological Society of America 94: 592–595. doi: 10.1603/0013-8746

[B54] Yeates DK Seago A Nelson L Cameron SL Joseph L Trueman JWH (2011) Integrative taxonomy, or iterative taxonomy? Systematic Entomology 36: 209–216. doi: 10.1111/j.1365-3113.2010.00558.x

[B55] Yee WL Sheets HD Chapman PS (2011) Analysis of surstylus and aculeus shape and size using geometric morphometrics to discriminate *Rhagoletis pomonella* and *Rhagoletis zephyria* (Diptera: Tephritidae). Annals of the Entomological Society of America 104: 105–114. doi: 10.1603/AN10029

[B56] Zelditch ML Swiderski DL Sheets HD Fink WL (2004) Geometric Morphometrics for Biologists: A Primer. Elsevier, Academic Press, New York, 436 pp.

